# Sexual orientation discrimination and eating disorder symptoms in early adolescence: a prospective cohort study

**DOI:** 10.1186/s40337-024-01157-y

**Published:** 2024-11-29

**Authors:** Jason M. Nagata, Thang Diep, Christiane K. Helmer, Sydnie K. Domingue, Abubakr A. Al-Shoaibi, Julia H. Raney, Kyle T. Ganson, Alexander Testa, Jinbo He, Claire D. Brindis, Fiona C. Baker

**Affiliations:** 1 Department of Pediatrics, University of California, San Francisco, 550 16th Street, 4th Floor, Box 0503, San Francisco, CA 94143 USA; 2 Factor-Inwentash Faculty of Social Work, University of Toronto, 246 Bloor St W, Toronto, ON M5S 1V4 Canada; 3 Department of Management, Policy and Community Health, University of Texas Health Science Center at Houston, 7000 Fannin, Suite 1706, Houston, TX 77030 USA; 4 Division of Applied Psychology, School of Humanities and Social Science, The Chinese University of Hong Kong, 2001 Longxiang Boulevard, Longgang District, Shenzhen, Guangdong China; 5 Philip R. Lee Institute for Health Policy Studies, University of California, San Francisco, 490 Illinois St, San Francisco, CA 94158 USA; 6 Center for Health Sciences, SRI International, 333 Ravenswood Ave, Menlo Park, CA 94025 USA; 7 School of Physiology, University of the Witwatersrand, 1 Jan Smuts Ave, Braamfontein, Johannesburg, 2017 South Africa

**Keywords:** Eating disorder, Disordered eating, Adolescence, LGBTQ, LGBT, Gay, Lesbian, Bisexual, Sexual orientation, Discrimination

## Abstract

**Background:**

Sexual orientation discrimination increases the risks of negative health outcomes for sexual minorities. Previous studies have found increased rates of eating disorder symptoms in sexual minority individuals, which is attributable to minority stress and discrimination that they experience. Emerging research suggests relationships between sexual orientation discrimination and eating disorder symptoms. However, there is a lack of studies focusing on early adolescents. The objective of this study was to determine prospective associations between discrimination based on sexual orientation and eating disorder symptoms in a national sample of 10–13-year-old early adolescents in the U.S.

**Methods:**

We examined prospective data from Year 2 (2018–2020) and Year 3 (2019–2021) of the Adolescent Brain Cognitive Development (ABCD) Study (N = 8976). Multiple logistic regression analyses were used to estimate associations between self-reported experiences of sexual orientation discrimination in Year 2 and eating disorder symptoms in Year 3, adjusting for potential confounders, including eating disorder symptoms in Year 2. Sexual orientation discrimination was assessed based on the Perceived Discrimination Scale, which measures adolescents’ perception of being treated unfairly based on various sociodemographic characteristics. Eating disorder symptoms were based on the parent-reported Kiddie Schedule for Affective Disorders and Schizophrenia (KSADS-5).

**Results:**

In this demographically diverse sample of early adolescents (N = 8976, age range 10–13 years at Year 2), 5.5% of adolescents reported sexual orientation discrimination in Year 2. The prevalence of parent-reported eating disorder symptoms in Year 3 varied from 1.0 to 8.3%. In the adjusted models, sexual orientation discrimination was prospectively associated with worry about weight gain (adjusted odds ratio [aOR] 2.33, 95% confidence interval [CI] 1.15–4.69) and self-worth tied to weight (aOR 1.60, 95% CI 1.01–2.53) one year later.

**Conclusions:**

Early adolescents who have experienced sexual orientation discrimination have higher odds of experiencing eating disorder symptoms, particularly worrying about weight gain and tying self-worth to weight. Clinicians may consider screening for sexual orientation discrimination and providing affirmative, trauma-informed care when evaluating and treating even younger sexual minority adolescents for eating disorder symptoms.

**Supplementary Information:**

The online version contains supplementary material available at 10.1186/s40337-024-01157-y.

## Plain language summary

Sexual orientation discrimination increases the likelihood of adverse health effects for sexual minorities. Prior research has identified increased rates of eating disorder symptoms among sexual minority individuals, linked to minority stress and discrimination they endure. Although recent studies suggest associations between sexual orientation discrimination and eating disorder symptoms, there is a gap in research concerning early adolescents. In this large, demographically diverse, national study of 8,976 adolescents aged 10–13 years, we found that early adolescents who have experienced sexual orientation discrimination are more likely to worry about weight gain and tie their self-worth to weight one year later. These findings support the Minority Stress Theory, which describes how discrimination, stigmatization, and internalized negative beliefs may contribute to worse mental health outcomes and maladaptive health behaviors, including eating disorder symptoms. Clinicians can screen for sexual orientation discrimination and provide affirmative, equity-focused care when evaluating even younger sexual minority patients for eating disorder symptoms.

**Supplementary Information:**

The online version contains supplementary material available at 10.1186/s40337-024-01157-y.

## Introduction

The prevalence of eating disorders has increased in recent years, specifically in adolescents [1]. Adolescence is a critical developmental period for the risk of eating disorders [2], and eating disorder symptoms often materialize during this time [2, 3]. Eating disorder symptoms, such as distress from binge eating, self-induced weight loss, intense fear of gaining weight, and self-worth tied to weight, are risk factors for developing full-threshold eating disorders [4]. Given that eating disorders are associated with adverse psychological and physical health outcomes like depression and multi-organ dysfunction [5–7], early identification and intervention of eating disorders and eating disorder symptoms are crucial for minimizing health consequences and reducing morbidity in adolescents [8].

Sexual minority adults and adolescents—those who identify as gay, lesbian, or bisexual, or who are attracted to or have sexual contact with individuals of the same gender [9]—are at higher risk of developing disordered eating behaviors, in comparison to their heterosexual peers [10]. For instance, nationally representative data of 9th to 12th-grade students found that sexual minority adolescent girls have 1.5 times the odds of using diet pills and 1.77 times the odds of purging, while sexual minority adolescent boys have more than twice the odds of fasting compared to their heterosexual sex-matched peers [11]. Another study of high school students in Massachusetts found that nearly one-third of sexual minority youth reported unhealthy weight control behaviors such as fasting for more than 24 h, using diet pills, or vomiting [12]. Additionally, sexual minority males are more than four times and sexual minority females are more than twice as likely to engage in such behaviors relative to their heterosexual peers [12]. Prior research established a strong groundwork for examining the prevalence of eating disorder symptoms in sexual minority adolescents, yet few studies investigated the experiences of early adolescents [11, 12]. One study that included early adolescents found that sexual orientation disparities in purging and binge eating were evident at the youngest ages and persisted through adolescence, but the data is now over a decade old, and the sample included participants ages 12–23 years old [13].

Moreover, a growing body of literature shows that societal pressures and stigmatization may be one underlying reason for the high prevalence of disordered eating in sexual minority adolescents [14, 15]. Sexual minority early adolescents are more likely to experience interpersonal discrimination because of their sexual orientation compared to their non-sexual minority peers [16, 17]. According to the Minority Stress Theory, sociopolitical factors and conditions perpetuate both internal (e.g., internalized homophobia, identity concealment) and external (e.g., discrimination, stigmatization, violence) stressors that disproportionately impact sexual minority individuals [18, 19]. These stressors create a hostile and unsafe environment that may worsen both physical and mental health outcomes [18, 19]. For instance, sexual orientation discrimination can serve as a minority stressor that contributes to mental health burdens, body dissatisfaction, and low self-esteem, which can predispose the development of eating disorder symptomatology across all age groups [17, 20, 21].

Our previous research showed that the prevalence of sexual orientation discrimination was highest among sexual minority adolescents (37.4%) [22]. However, approximately 3% of heterosexual adolescents still reported sexual orientation discrimination [22]. This may occur because heterosexual adolescents who deviate from the societal expectations of gender roles and sexual identity can be perceived differently by their peers and might be teased based on these perceptions. As a result, these individuals may internalize this teasing as sexual orientation discrimination. Consequently, heterosexual adolescents may still experience the negative effects associated with sexual orientation discrimination, so it is important to explore how sexual orientation discrimination may impact eating disorder symptoms within this group. Despite the burden of sexual orientation discrimination in adolescents, few studies have linked sexual orientation discrimination specifically with eating disorder symptoms, particularly in a large prospective cohort of early adolescents.

In the present study, we aimed to examine the prospective association between sexual orientation discrimination and DSM-5 eating disorder symptoms from a large, nationally diverse sample of early adolescents in the U.S. We hypothesized that sexual orientation discrimination would be prospectively associated with eating disorder symptoms in early adolescents.

## Methods

### Study sample

We examined prospective data from the Adolescent Brain Cognitive Development (ABCD) Study, a longitudinal study of brain development and health in the U.S. In 2016–2018 (baseline; Year 0), 11,875 children mostly aged 9–10 years were recruited from 21 demographically diverse sites across the nation. These participants were recruited mainly through elementary schools and were selected via stratified, probability sampling of U.S. schools within the 21 catchment areas. Institutional review board approval was granted from the University of California, San Diego, and at each of the 21 study sites. Written assent was provided by participants, and written informed consent was provided by caregivers.

Data analyzed in the present study were from the ABCD 5.1 release from Year 2 (2018–2020) and Year 3 (2019–2021), with early adolescents aged 11–14 years at Year 3. Evaluation of youth-reported eating disorder symptoms was only performed at the Year 2 assessment, so we opted to use parent-reported eating disorder symptoms instead to conduct a prospective analysis. Out of the participants enrolled in the study, 2024 were missing parent-reported eating disorder symptoms data, 828 were missing sexual orientation discrimination data, and 134 were missing sociodemographic data (Additional File 1: Appendix A). We did not exclude majority groups in the analyses because they can also face discrimination. For instance, approximately 3% of heterosexuals reported discrimination based on sexual orientation [22]. Our inclusion criteria yielded a final analytical sample of 8976 participants.

### Independent variable: sexual orientation discrimination (year 2)

Sexual orientation discrimination in Year 2 was measured from the Perceived Discrimination Scale [23, 24], which measures adolescents’ perception of being treated unfairly based on various sociodemographic characteristics. Participants were asked, “In the past 12 months, have you felt discriminated against because someone thought you were gay, lesbian, or bisexual?” Response options were “Yes,” “No,” “Don’t know,” and “Refuse to answer,” and those who answered with the latter two responses were excluded from the analysis (3.0% and 0.4%, respectively).

### Dependent variable: symptoms of eating disorders (year 3)

The ABCD study uses the Kiddie Schedule for Affective Disorders and Schizophrenia (KSADS-5), a validated computerized tool for diagnostically categorizing child and adolescent mental health issues according to DSM-5 criteria, to evaluate eating disorder symptoms [25]. The KSADS-5 measured the frequency, duration, and characteristics of these symptoms. We used parent-reported eating disorder symptoms at Year 3. Participants’ parents were asked, “Does your child feel like his or her self-worth is tied to his or her weight?” and “In the past two weeks, how often has your child been preoccupied with gaining weight or worrying a lot about being fat?” Additionally, they were asked about their child’s behaviors, including compensatory behaviors to lose weight, binge eating, and distress with binge eating. Compensatory behaviors included eating foods with minimal calories, exercising a lot, throwing up, and taking water pills, laxatives, or diet pills. Due to the low prevalence of these behaviors in the study sample, participants who reported any of these actions were classified as engaging in compensatory behaviors to lose weight.

### Covariates

We adjusted for the following covariates: age, sex (female or male), sexual orientation (heterosexual, maybe gay/bisexual, gay/bisexual, don’t understand the question, or refused to answer), race and ethnicity (Asian, Black, Latino/Hispanic, Native American, White, or other), household income (grouped into six categories reflecting the US median household income), and highest parental education (high school or less or college education or more). Prior studies demonstrated that these variables have been associated with sexual orientation discrimination and/or eating disorder symptoms [26–28]. Furthermore, race was controlled for because racial identity may intersect with sexual orientation, supporting the intersectionality framework in that the intersection of marginalized identities may contribute to health disparities [29]. Household income and parental education are both proxies of socioeconomic status and can affect resources and support, potentially influencing experiences of discrimination and the development of eating disorder symptoms. Additionally, the ABCD study site was included as a covariate to account for potential geographic variation. All covariates were from Year 2 except for sexual orientation, which was from Year 3 because it was more reflective of the adolescents’ true sexual orientation since more of them had come out by Year 3. At the Year 3 visit, participants were asked “Are you gay or bisexual?” with response options of “Yes,” “Maybe,” “No,” “Do not understand,” and “Refuse to answer” from the background section of the Kiddie Schedule of Affective Disorders and Schizophrenia [9]. Each respective symptom of an eating disorder as reported by the parent from Year 2 was included as a covariate.

### Statistical analysis

Data analyses were performed using Stata 18 (StataCorp LLC). Descriptive statistics, including means, standard deviations, and percentages, were calculated. Multiple logistic regression analyses were used to estimate associations between sexual orientation discrimination in Year 2 as the independent variable and the presence of eating disorder symptoms in Year 3 (worry about weight gain, self-worth tied to weight, inappropriate compensatory behavior to prevent weight gain, binge eating, and distress with binge eating) as the dependent variable, adjusting for covariates including the respective eating disorder symptom in Year 2. Sex-stratified analyses were also conducted given potential sex differences in sexual orientation discrimination and eating disorder symptoms. Interaction between sexual orientation and sexual orientation discrimination on eating disorder symptoms was tested for. Propensity weights were applied to yield representative estimates based on the American Community Survey from the US Census [30].

## Results

Table 1 reports the descriptive characteristics of the 8976 adolescents included in our analytical sample. The sample was approximately matched by sex (48.5% female), included sexual minority adolescents (8.5% identified as gay/bisexual), and was racially and ethnically diverse (51.5% racial/ethnic minority). The prevalence of sexual orientation discrimination in Year 2 was 5.5% (26.4% among gay/bisexual, 8.4% among maybe gay/bisexual, 3.1% among heterosexual). The prevalence of eating disorder symptoms in Year 3 varied from 1.0 to 8.3%.

**Table 1:** Sociodemographic characteristics and prevalence of sexual orientation discrimination and eating disorder symptoms of Adolescent Brain Cognitive Development (ABCD) Study participants (N = 8976)

Sociodemographic characteristics (Year 2)	Mean (SD) / %
Age (years)	12.0 (0.7)
*Sex*	
Female	48.5%
Male	51.5%
*Sexual orientation (Year 3)*	
Heterosexual	83.5%
Gay/bisexual	8.5%
Maybe gay/bisexual	5.4%
Don’t understand the question	1.3%
Refuse to answer	1.3%
*Race and ethnicity*	
Asian	5.3%
Black	14.6%
Latino/Hispanic	19.6%
Native American	3.2%
Other^a^	1.4%
White	55.9%
*Household income*	
$24,999 or less	13.3%
$25,000 to $49,999	16.7%
$50,000 to $74,999	16.6%
$75,000 to $99,999	14.5%
$100,000 to $199,999	28.7%
$200,000 and greater	10.3%
*Parent’s highest education*	
High school education or less	11.4%
College education or more	88.6%
Sexual orientation discrimination	5.5%
*Parent-reported eating disorder symptoms (Year 3)*	
Worry about weight gain	1.0%
Self-worth tied to weight	4.2%
Inappropriate compensatory behaviors to prevent weight gain	8.3%
Binge eating	3.9%
Distress with binge eating	2.5%

ABCD sampling weights were applied to yield representative estimates based on the American community survey from the US census

^a^This subcategory was listed as “other” but with no specific racial and ethnic groups defined, although write-ins were allowed

Prospective associations between sexual orientation discrimination and eating disorder symptoms are presented in Table 2. After adjusting for covariates, including respondent sexual orientation, we found that sexual orientation discrimination was prospectively associated with worry about weight gain (adjusted odds ratio [aOR] 2.33, 95% confidence interval [CI] 1.15–4.69) and self-worth tied to weight (aOR 1.60, 95% CI 1.01–2.53) one year later. Sexual orientation discrimination was not significantly associated with inappropriate compensatory behaviors to prevent weight gain, binge eating, and distress with binge eating.

**Table 2:** Prospective associations between sexual orientation discrimination and eating disorder symptoms in the Adolescent Brain Cognitive Development (ABCD) Study (N = 8976)

	Sexual orientation discrimination (year 2, independent variable)			
Eating disorder symptoms (Year 3, dependent variable)	Unadjusted OR (95% CI)	p	Adjusted OR (95% CI)^a^	p
Worry about weight gain	**3.05 (1.55, 6.01)**	**0.005**	**2.33 (1.15, 4.69)**	**0.018**
Self-worth tied to weight	**2.19 (1.48, 3.25)**	**0.001**	**1.60 (1.01, 2.53)**	**0.044**
Inappropriate compensatory behaviors to prevent weight gain	**1.62 (1.16, 2.27)**	** < 0.001**	1.25 (0.83, 1.89)	0.286
Binge eating	1.44 (0.90, 2.29)	0.125	1.00 (0.57, 1.78)	0.994
Distress with binge eating	1.67 (0.96, 2.91)	0.069	1.16 (0.58, 2.30)	0.681

Bold indicates *p* < 0.05. Models represent the abbreviated output from 10 logistic regression models with sexual orientation discrimination in year 2 as the independent variable and eating disorder symptoms in year 3 as the dependent variable. ABCD propensity weights were applied to yield representative estimates based on the American community survey from the US census

^a^Adjusted for eating disorder symptoms at year 2, age, sex, sexual orientation, race and ethnicity, household income, parent education, and study site

Additional File 2: Appendix B shows prospective sex-stratified analyses between sexual orientation discrimination and eating disorder symptoms. In female adolescents, sexual orientation discrimination was prospectively associated with worry about weight gain (aOR 2.72, 95% CI 1.20–6.15) one year later, after adjusting for covariates. In male adolescents, sexual orientation discrimination was prospectively associated with distress with binge eating (aOR 2.50, 95% CI 1.12, 5.57) and self-worth tied to weight (aOR 2.05, 95% CI 1.06, 3.94) one year later, after adjusting for covariates. The interaction between sexual orientation and sexual orientation discrimination on the association with eating disorder symptoms was not significant.

## Discussion

In a demographically diverse national sample in the U.S., our study found that sexual orientation discrimination in early adolescents was associated with over twice the odds of worrying about weight gain and higher odds of tying one’s self-worth to one’s weight. In adolescent boys, sexual orientation discrimination was associated with distress with binge eating and self-worth tied to weight. In adolescent girls, sexual orientation discrimination was associated with fear of weight gain.

The results of our study add to the literature by investigating the prospective associations between sexual orientation discrimination and DSM-5 eating disorder symptoms in early adolescents whose developmental period is vulnerable to developing unhealthy and disordered eating behaviors [2, 3]. Prior research found sexual orientation disparities in eating disorders and disordered eating behaviors, with higher prevalence of sexual minority adolescents engaging in weight control behaviors such as fasting, using diet pills, or vomiting [11, 12, 31]. In addition to the high prevalence of eating disorder symptoms in sexual minority adolescents, our findings suggest that there is a prospective association between the experience of discrimination because of sexual orientation and subsequent eating disorder symptoms in early adolescents.

The higher risks of eating disorder symptoms in early adolescents who have experienced sexual orientation discrimination may be due to elevated sexual minority stress levels. Although the exact mechanisms by which sexual orientation discrimination may influence the development of eating disorder symptoms remain unclear, the Minority Stress Theory describes how discrimination, stigmatization, and internalized negative beliefs may contribute to worse mental health outcomes and maladaptive health behaviors [16–19]. For instance, research on lesbian adults found that sexual minority stress was associated with social anxiety [20], which has been linked to more body shame and binge eating [32, 33]. A systematic review on the role of minority stress in disordered eating also found that shame, body shame, or negative affect are important mediators between minority stress and disordered eating in adults [34]. Sexual orientation discrimination may also undermine sexual minority individuals’ ability to recognize and regulate their emotions by serving as a form of traumatic invalidation [35]. Emotional dysregulation can then predispose sexual minority individuals to adopt maladaptive behaviors as a means of coping with their emotions and fulfilling their needs [35], which may explain the binge eating distress seen among adolescent boys in our study who previously experienced sexual orientation discrimination. Moreover, stressors that target sexual orientation may uniquely impact youth mental health with one study showing that mental health burden was more common in early adolescents who reported experiencing discrimination based on their sexual orientation even when accounting for other interpersonal stressors not related to sexual orientation [17].

We found notable sex differences in some of the associations. Adolescents boys may face more victimization and discrimination due to their sexual orientation [36]. The association between sexual orientation discrimination and distress with binge eating in boys may be attributed to the higher prevalence of binge-eating behaviors in early adolescent boys compared to girls [26]. Girls have more fear of weight gain than boys, which reflects the feminine body ideal of thinness [37].

Findings from the present study hold significant implications for clinical practices, the education sector, public health, and public policy. For example, clinicians may consider screening for eating disorder behaviors in even their youngest adolescent patients who have experienced discrimination based on their sexual orientation. They should also integrate trauma-informed principles and a strength-based approach when evaluating and treating them for eating disorder symptoms [38]. However, given that clinicians play a relatively brief role in the lives of their patients, public policymakers should consider the larger role that schools play in adolescent development and their ability to ascertain that many sexual minority youth may experience stigma and discrimination at school [39]. In response, schools can bolster protective factors such as LGBTQ-inclusive curricula, supportive educators, and anti-bullying policies that have been shown to foster school connectedness and perceptions of personal safety [40], as well as potentially playing a role in referring adolescents experiencing sexual orientation discrimination. These strategies may decrease the minority stress level experienced by sexual minority adolescents [16], which in turn may decrease the risk of developing eating disorder symptomatology [20].

This study has several limitations. First, the Perceived Discrimination Scale focused on sexual orientation discrimination by other students, not peers more broadly, which may have missed perpetrators they interacted with outside of school. Additionally, this study used parent-reported eating disorder symptoms since evaluation of youth-reported eating disorder symptoms was not performed at Year 3. Although parents are important reporters of eating disorders in adolescents since children often have limited insight into their eating behaviors [41, 42], there tends to be low concordance between parent and youth reports of disordered eating behaviors [41–43]. Specifically, a study demonstrated parents may provide lower estimates of behavioral symptoms of eating disorders [44]. Moreover, measures in our study relied on self-reported responses to survey questions and may be subjected to reporting bias. Due to differences in sociodemographic characteristics between participants included and excluded in the analysis, it limits our ability to generalize the results of our study. We also cannot infer causality given the observational nature of the study. Due to limited cell size and insufficient power, we were not able to analyze associations stratified by sexual orientation categories. Future research could examine these associations with larger samples of sexual minorities.

Despite these limitations, our study has several strengths. To our knowledge, this is the first study to examine the prospective association between sexual orientation discrimination and eating disorder symptoms in early adolescents. Other similar studies have only examined pooled cross-sectional data [11, 12]. Our results support the Minority Stress Theory [16, 18] in that sexual orientation discrimination could be one factor that contributes to disparities in disordered eating behaviors [14, 27].

## Conclusion

Adolescents who have experienced sexual orientation discrimination have higher odds of experiencing eating disorder symptoms one year later, particularly worrying about weight gain and tying self-worth to weight. This prospective study underscores the importance of addressing sexual orientation discrimination and building supportive environments for sexual minority youth in the prevention and intervention of eating disorders in early adolescence. Future research can investigate the mechanisms by which sexual orientation discrimination may affect the development of eating disorders even in younger adolescents and treatment options that incorporate coping strategies to effectively manage sexual orientation discrimination.

## Supplementary Information

Additional file 1.

Additional file 2.

## Data Availability

Data used in the preparation of this article were obtained from the ABCD Study (https://abcdstudy.org), held in the NIMH Data Archive (NDA).

## Abbreviations

ABCD: Adolescent Brain Cognitive Development Study
KSADS-5: Kiddie Schedule for Affective Disorders and Schizophrenia
