# Supplementary material for: Sexual orientation discrimination and eating disorder symptoms in early adolescence: a prospective cohort study
Source: J Eat Disord. 2024 Nov 29;12:196. doi: 10.1186/s40337-024-01157-y (PMC11606175; doi:10.1186/s40337-024-01157-y)
Supplement: Supplementary file 1 — Additional file 1. [file 40337_2024_1157_MOESM1_ESM.docx]

| Appendix A. Comparison of the sociodemographic characteristics of the Adolescent Brain Cognitive Development (ABCD) Study participants included vs. excluded in the analysis | | | |
| --- | --- | --- | --- |
| Sociodemographic characteristics | Included (n=8,976) | Excluded (n=2,986) | p |
| Age (years) | 12.0 (0.7) | 12.1 (0.7) | 0.004 |
| Sex (%) |  |  | 0.269 |
| Female | 48.5% | 49.8% |  |
| Male | 51.5% | 50.2% |  |
| Sexual orientation (%) |  |  | <0.001 |
| Heterosexual | 83.5% | 75.2% |  |
| Gay/bisexual | 8.5% | 11.3% |  |
| Maybe gay/bisexual | 5.4% | 5.9% |  |
| Don't understand the question | 1.3% | 4.5% |  |
| Refuse to answer | 1.3% | 3.1% |  |
| Race and ethnicity (%) |  |  | <0.001 |
| Asian | 5.3% | 6.1% |  |
| Black | 14.6% | 25.7% |  |
| Latino/Hispanic | 19.6% | 21.5% |  |
| Native American | 3.2% | 3.0% |  |
| Other | 1.4% | 1.7% |  |
| White | 55.9% | 42.0% |  |
| Household income (%) |  |  | <0.001 |
| $24,999 or less | 13.3% | 21.2% |  |
| $25,000 to $49,999 | 16.7% | 22.4% |  |
| $50,000 to $74,999 | 16.6% | 13.7% |  |
| $75,000 to $99,999 | 14.5% | 10.3% |  |
| $100,000 to $199,999 | 28.7% | 26.5% |  |
| $200,000 or greater | 10.3% | 5.8% |  |
| Parent's highest education (%) |  |  | <0.001 |
| High school education or less | 11.4% | 26.2% |  |
| College education or more | 88.6% | 73.8% |  |
