# Supplementary material for: Sexual orientation discrimination and eating disorder symptoms in early adolescence: a prospective cohort study
Source: J Eat Disord. 2024 Nov 29;12:196. doi: 10.1186/s40337-024-01157-y (PMC11606175; doi:10.1186/s40337-024-01157-y)
Supplement: Supplementary file 2 — Additional file 2. [file 40337_2024_1157_MOESM2_ESM.docx]

| Appendix B. Prospective associations between sexual orientation discrimination and eating disorder symptoms in the Adolescent Brain Cognitive Development (ABCD) Study, stratified by sex | | | | |
| --- | --- | --- | --- | --- |
|  | Female | | Male | |
| **Eating disorder symptom** | Adjusted OR (95% CI) | p | Adjusted OR (95% CI) | p |
| Worry about weight gain | **2.72 (1.20, 6.15)** | **0.016** | 2.43 (0.60, 9.84) | 0.213 |
| Self-worth tied to weight | 1.46 (0.82, 2.60) | 0.197 | **2.05 (1.06, 3.94)** | **0.032** |
| Inappropriate compensatory behaviors to prevent weight gain | 1.25 (0.76, 2.04) | 0.377 | 1.26 (0.64, 2.46) | 0.501 |
| Binge eating | 0.55 (0.22, 1.38) | 0.204 | 1.70 (0.82, 3.52) | 0.150 |
| Distress with binge eating | 0.35 (0.12, 1.04) | 0.058 | **2.50 (1.12, 5.57)** | **0.025** |
| Bold indicates p<0.05. Models represent the abbreviated output from the logistic regression models with adjustment for parent-reported eating disorder symptoms at Year 2, age, sexual orientation, race and ethnicity, household income, parent education, and site. ABCD propensity weights were applied to yield representative estimates based on the American Community Survey from the US Census. | | | | |
